# A Bayesian Approach for Estimating the Survivor Average Causal Effect When Outcomes Are Truncated by Death in Cluster-Randomized Trials

**DOI:** 10.1093/aje/kwad038

**Published:** 2023-02-17

**Authors:** Guangyu Tong, Fan Li, Xinyuan Chen, Shashivadan P Hirani, Stanton P Newman, Wei Wang, Michael O Harhay

**Keywords:** always-survivors, Bayesian estimation, cluster-randomized trials, counterfactual outcomes, death truncation, principal stratification, quality of life, survivor average causal effect

## Abstract

Many studies encounter clustering due to multicenter enrollment and nonmortality outcomes, such as quality of life, that are truncated due to death—that is, missing not at random and nonignorable. Traditional missing-data methods and target causal estimands are suboptimal for statistical inference in the presence of these combined issues, which are especially common in multicenter studies and cluster-randomized trials (CRTs) carried out among the elderly or seriously ill. Using principal stratification, we developed a Bayesian estimator that jointly identifies the always-survivor principal stratum in a clustered/hierarchical data setting and estimates the average treatment effect among them (i.e., the survivor average causal effect (SACE)). In simulations, we observed low bias and good coverage with our method. In a motivating CRT, the SACE and the estimate from complete-case analysis differed in magnitude, but both were small, and neither was incompatible with a null effect. However, the SACE estimate has a clear causal interpretation. The option to assess the rigorously defined SACE estimand in studies with informative truncation and clustering can provide additional insight into an important subset of study participants. Based on the simulation study and CRT reanalysis, we provide practical recommendations for using the SACE in CRTs and software code to support future research.

## Abbreviations

CRT: cluster-randomized trial
ICC: intracluster correlation coefficient
LMM: linear mixed model
MCMC: Markov chain Monte Carlo
SACE: survivor average causal effect
WSD: Whole Systems Demonstrator

Outcomes such as quality of life are frequently measured nonmortality outcomes used to assess general health, recovery, and the impact of medical interventions (1–3). In studies with nontrivial mortality, such as those conducted among the elderly or persons with critical and serious illnesses, patient-centered outcome measures often cannot be captured for a sizeable proportion of participants who die during the study period (4, 5). Nonmortality outcomes with missing data due to death raise conceptual and empirical issues. First, death itself is an outcome of interest. Second, because the nonmortality outcome is empirically unmeasured (i.e., undefined) among persons who die, imputation approaches may not appeal to certain stakeholders (6–8). Relatedly, composite outcomes require that some subjective valuation be used concerning what value should be used for those who die (9, 10). Further, from a causal inference perspective, many strategies may not provide estimates with a clear causal interpretation under the counterfactual outcomes framework (11–13) because surviving study participants under treatment and control conditions can be systematically different, which obscures the target estimand.

**Figure 1:**
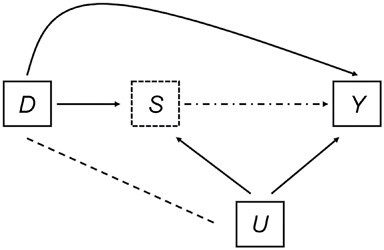
Conceptual diagram for the problem of an outcome truncated by death. *D*, treatment; *S*, survivor status; *Y*, outcome; *U*, unobserved variables. The solid lines with arrows show the directed effect; the dashed line shows a potential association; and the dotted-dashed line with an arrow indicates that the survival status may prevent the outcome from being observed. Adapted from Suzuki (14).

Adapted from Suzuki (14), Figure 1 portrays what is often termed the “truncation-by-death” problem. In this setting, the survivor status (*S*) and quality-of-life outcome (*Y*) of an individual can be influenced by the treatment (*D*), as well as by an unobserved variable (*U*). The unobserved variable (*U*) can also be potentially associated with the treatment (*D*), but the association would be less likely if the treatment were assigned in a randomized trial setting. Under the counterfactual outcomes framework, there is considerable literature on estimating causal effects using principal stratification (15–19). This framework has been applied to study nonmortality outcomes in individually randomized trials (4, 18), as well as to address protocol compliance, patient encouragement, and other problems in public health and social science settings (e.g., the effect of job training programs and behavioral health interventions) (16, 17, 20, 21). Principal stratification seeks to identify strata of patients by their preexposure characteristics. The stratum of primary interest includes patients who would always survive through the end of the trial period regardless of treatment assignment. The effect of an intervention in this stratum of “always-survivors” is termed the survivor average causal effect (SACE). The SACE is a meaningful estimand because, without additional assumptions, only the always-survivors have both counterfactual outcomes well-defined. Thus, the SACE avoids survivor bias (i.e., observed and unobserved characteristics of survivors in treatment and control are likely different) and summarizes the treatment effect without needing additional assumptions about the counterfactual outcomes for the nonsurvivors (5, 10, 22, 23). However, the always-survivors target population is not directly observed; that is, not all participants have a definitive stratum membership, but principal strata must be identified to estimate the SACE. Bayesian inference is particularly attractive for this purpose, since the posterior strata membership can be updated through a Markov chain Monte Carlo (MCMC) algorithm, while posterior predictive distributions for the SACE can be conditional on the strata membership (15, 16).

Herein, we extend and use principal stratification to estimate the SACE using Bayesian inference in cluster-randomized trials (CRTs) with death truncation. In contrast to individual-level randomization, CRTs randomize groups of individuals to treatments (24, 25). Cluster-level randomization is used when the intervention is designed for a system-level improvement or when randomization is not feasible at the individual level (see Turner et al. (25) for a review). As a result, the outcome observations are often more similar within clusters than between clusters, causing a positive intracluster correlation that must be accounted for at the analysis stage to avoid inflated type I error. To estimate the SACE in CRTs, we developed a Bayesian approach that leverages the baseline covariates to predict the counterfactual survivor status and the counterfactual outcomes. As we explicate in the Methods section, our approach includes both a principal stratification model and an outcome model, for which we developed an iterative sampling algorithm to estimate the model parameters jointly, and hence the SACE in CRTs.

## METHODS

### Causal framework and assumptions

We consider the counterfactual outcome framework, where the causal effect is defined as the difference between the 2 counterfactual outcomes averaged across a common population (12, 13). We assume 1) the stable unit treatment value assumption by which patients receive no different forms or versions of treatment and that no interference exists and 2) that the treatment is randomized at the cluster level and is independent of both the potential survivor status and the counterfactual outcome of all individuals in each cluster. In a CRT with $I$ clusters and ${n}_i$ individuals in each cluster, let us denote the cluster-level treatment and control assignment as ${D}_i=1,0$, respectively, where $i=1,\dots, .I$For the *j* th individual ($j=1,\dots, {n}_j$) in the *i *th cluster, we define $\{{Y}_{ij}(1),{Y}_{ij}(0)\}$ as the counterfactual outcomes for each individual under treatment and control conditions. We are typically interested in the average causal effect, $\delta =E({Y}_{ij}(1)-{Y}_{ij}(0))$, if the counterfactual outcomes are well-defined for the entire trial population. However, when outcomes are truncated, the average causal effect can only be defined for a subset of patients. Estimating the average causal effect in a meaningful subgroup in this setting involves 1) using the covariates and survival status to identify the potentially unobserved always-survivor stratum and 2) comparing counterfactual outcomes within the always-survivor stratum under treatment and control conditions. Below we describe models for each of these components.

### Principal strata model for survivor status

We define ${S}_{ij}$ as the observed survival status of a patient, with ${S}_{ij}=1$ indicating survival and ${S}_{ij}=0$ indicating death. Under the potential outcomes framework, the joint values of potential survival status produce 4 strata. These include:

1. *Always-survivors* (${S}_{ij}(1)={S}_{ij}(0)=1$): patients who will survive until the end of the study regardless of their treatment status.
2. *Protected individuals* (${S}_{ij}(1)=1$, ${S}_{ij}(0)=0$): patients who will survive only under treatment.
3. *Harmed individuals* (${S}_{ij}(1)=0,{S}_{ij}(0)=1$): patients who will survive only under control conditions.
4. *Never-survivors* (${S}_{ij}(1)={S}_{ij}(0)=0$): patients who will not survive regardless of treatment.

We make an additional assumption of *monotonicity* such that the treatment does not lead to worse survival, and thus the harmed stratum is assumed away (17, 26). Monotonicity is a plausible assumption in trials when interventions are carefully piloted for safety considerations. However, we acknowledge that an intervention can lead to worse survival in some patients, and in such instances, the monotonicity assumption will be questionable. In scenarios where the proportion of a harmed stratum is close to 0, modeling the harmed stratum can also lead to computational challenges, in which case the monotonicity assumption represents a practical consideration for model-fitting. In our motivating trial, we considered the harmed patients to be rare (if not nonexistent) and undertook the analysis assuming monotonicity. Potential approaches to relaxing this assumption are discussed below. Under monotonicity, the principal strata membership is observed for survivors in the control group and the deceased in the treatment group, but it is unknown for survivors in the treatment group or the deceased in the control group. Table 1 presents the strata membership attribution for each observed data group under monotonicity. Baseline covariates play a critical role in identifying the principal strata. Suppose ${G}_{ij}=\left\{00,10,11\right\}$ indicates principal strata membership, where ${G}_{ij}=00$ for never-survivors, ${G}_{ij}=10$ indicates protected individuals, and ${G}_{ij}=11$ indicates always-survivors. Assuming ${X}_{ij}$ as a covariate vector with both cluster-level and individual-level covariates and further including an intercept, the principal strata can be modeled using multinomial logistic regression with ${G}_{ij}=11$ as the reference group:

$$\begin{align*} P({G}_{ij}=00)&=\frac{e^{\boldsymbol{X}_{ij}^T{\boldsymbol\beta} }}{1+{e}^{\boldsymbol{X}_{ij}^T{\boldsymbol\beta} }+{e}^{\boldsymbol{X}_{ij}^T{\boldsymbol\gamma} }};\\ P({G}_{ij}=10)&=\frac{e^{\boldsymbol{X}_{ij}^T\boldsymbol\gamma}}{1+{e}^{\boldsymbol{X}_{ij}^T{\boldsymbol\beta} }+{e}^{\boldsymbol{X}_{ij}^T\boldsymbol\gamma}};\\ P({G}_{ij}=11)&=\frac{1}{1+{e}^{\boldsymbol{X}_{ij}^T{\boldsymbol\beta} }+{e}^{\boldsymbol{X}_{ij}^T\boldsymbol\gamma}}. \end{align*}$$

**Table 1:** Data Elements and Principal Strata Membership Based on Observed Survivor Status and Treatment Status Under the Monotonicity Assumption

**Observed** **Group**	**Observed Treatment** **Status**	**Observed** **Survival** **Status**	**Observed Outcome** ^**a**^	**Unobserved Outcome**	**(Possible) Strata Membership Under Monotonicity**
${D}_i=1$ , ${S}_{ij}=1$	Yes	Yes	${Y}_{ij}={Y}_{ij}(1)$	${Y}_{ij}(0)$	Always-survivor or protected individual
${D}_i=1$ , ${S}_{ij}=0$	Yes	No	${Y}_{ij}={Y}_{ij}(1)$ = *	${Y}_{ij}(0)$	Never-survivor
${D}_i=0$ , ${S}_{ij}=1$	No	Yes	${Y}_{ij}={Y}_{ij}(0)$	${Y}_{ij}(1)$	Always-survivor
${D}_i=0$ , ${S}_{ij}=0$	No	No	${Y}_{ij}={Y}_{ij}(0)$ = *	${Y}_{ij}(1)$	Protected individual or never-survivor

^a^ An asterisk (*) indicates truncation by death.

Here, $\boldsymbol\beta$ and $\boldsymbol\gamma$ are *p*-dimensional regression coefficient vectors for never-survivors versus always-survivors and for the protected versus always-survivors, respectively; each component of $\boldsymbol\beta$ and $\boldsymbol\gamma$ is interpreted as the log odds ratio. To ensure numerical stability, we recommend choosing the (likely) largest stratum as the reference category for the multinomial logistic model.

In addition, cluster-level random intercepts can be added when principal strata membership is believed to be correlated due to cluster randomization. Alternatively, a nested probit model for the strata membership can be used (16); however, the regression coefficients are more challenging to interpret. Thus, we did not pursue the nested probit model further but derived its posteriors with a latent variable specification to support its use by interested readers (see Web Appendix 1, available at https://doi.org/10.1093/aje/kwad038).

### Models for potential outcomes

After defining the principal strata model, we specify counterfactual outcome models within each principal stratum. Specifically, only the always-survivors have well-defined counterfactual outcomes under both treatment and control conditions (${Y}_{ij}(0)$, ${Y}_{ij}(1)$), since they are not subject to truncation. Patients in protected strata have only 1 well-defined counterfactual outcome under treatment, but their counterfactual outcome under the control condition is truncated by death (${Y}_{ij}(0)=\ast$, ${Y}_{ij}(1)$). Among the always-survivors, each counterfactual outcome can be modeled as a function of covariates adjusting for clustering, whereas only 1 counterfactual outcome for each protected patient can be similarly modeled.

Now, let ${\boldsymbol\alpha}_1^{11}$, ${\boldsymbol\alpha}_0^{11}$, and ${\boldsymbol\alpha}_1^{10}$ be *p*-dimensional vectors of regression coefficients for covariates in these 3 groups: always-survivors in the treatment group (${G}_{ij}=11$, ${D}_i=1),$always-survivors in the control group (${G}_{ij}\!\!=\!\!11$, ${D}_i\!\!=\!\!0$), and protected individuals in the treatment group (${G}_{ij}=10$, ${D}_i= 1$). Let ${Y}_{ij}$ be the outcome where $(i,j)\in \left\{{S}_{ij}(D_i)=1\right\}$. Let ${\eta}_i$ be the cluster-level random effect following a normal distribution with mean 0 and variance ${\tau}^2$; let the residual error follow a normal distribution with a mean equal to 0 and a variance of ${\sigma}^2$. The assumed linear mixed models (LMMs) for the counterfactual outcomes can then be summarized as in Table 2. The random-effects term ${\eta}_i$ is required here to account for the intracluster correlation coefficient (ICC), $\rho =\frac{\tau^2}{\tau^2+{\sigma}^2}$, a quantity that is central to the design and analysis of CRTs, since ignoring the ICC in the outcome model leads to an inflated type I error rate (27). With the outcome model specified for always-survivors, the SACE can be defined as

$$\begin{align*} \delta =E({Y}_{ij}(1)-{Y}_{ij}(0)|{G}_{ij}=11). \end{align*}$$

**Table 2:** Outcome Model According to Principal Stratum and Counterfactual of Treatment Status

**Principal Stratum**	**Counterfactual**	
	**Treatment Group** **(** ${D}_i=1$ **)**	**Control Group** **(** ${D}_i=0$ **)**
Always-survivors (${G}_{ij}=11$)	${Y}_{ij}(1)=N\big({ {\textsf {X}}}_{ij}^T{\boldsymbol\alpha}_1^{11}+{\mathrm{\eta}}_i,{\mathrm{\sigma}}^2\big)$	${Y}_{ij}(0)=N\big({\textsf {X}}_{ij}^T{\boldsymbol\alpha}_0^{11}+{\mathrm{\eta}}_i,{\mathrm{\sigma}}^2\big)$
Protected individuals (${G}_{ij}=10$)	${Y}_{ij}(1)=N\big({\textsf {X}}_{ij}^T{\boldsymbol\alpha}_1^{10}+{\mathrm{\eta}}_i,{\mathrm{\sigma}}^2\big)$	
Never-survivors (${G}_{ij}=00$)		

We leverage the mixture modeling assumptions and the monotonicity to jointly estimate the strata membership probability for each individual and the counterfactual outcome model parameters. This approach produces the treatment effect for each always-survivor, which can be averaged over the always-survivor subpopulation to identify the SACE. Beyond the mixture modeling approach, we acknowledge that other structural assumptions can be used to identify the SACE even in the absence of monotonicity (see, for example, Hayden et al. (28) and Shepherd et al. (29)).

### Joint inference of the outcome model and the principal strata model

Posterior inference of the parameters in the principal strata model and the outcome model can be achieved through an MCMC algorithm. The algorithm is summarized in the Appendix, and detailed derivations for each step are presented in Web Appendix 1. The algorithm uses Gibbs sampling steps to update outcome regression model parameters, where conjugate priors of normal and inverse gamma distributions are specified (details are given in Web Appendix 1). The algorithm further implements a Metropolis-Hastings step for the principal stratification model. To increase the convergence speed for $\boldsymbol\beta$ and $\boldsymbol\gamma$, we use a random-walk Metropolis algorithm (30) that draws proposals from multivariate *t* distributions, $t({s}_{\beta }\boldsymbol{T}_{\beta})$ and $t({s}_{\gamma }\boldsymbol{T}_{\gamma})$, that center at the values of the previous iteration. The parameters ${s}_{\beta }$ and ${s}_{\gamma }$ scale the covariance to achieve optimal acceptance rates, and both $\boldsymbol{T}_{\beta }$ and $\boldsymbol{T}_{\gamma }$ are $p\times p$-dimensional component-specific scale matrices. We use the adaptive proposal approach by Haario et al. (31) to tune $\boldsymbol{T}_{\beta }$ and $\boldsymbol{T}_{\gamma }$ by utilizing empirical covariance from an extended burn-in. As indicated by the asterisks (*) in the Appendix, when the principal strata model also accounts for clustering (denoted as ${\chi}_i$ for the random intercept, where ${\chi}_i\sim N(0,{\phi}^2)$), it can be updated using the same approach as for $\boldsymbol\beta$ and $\boldsymbol\gamma$.

### Simulation study

We conducted a simulation study to validate our algorithm. Specifically, we simulated a 2-arm CRT with 1,500 individuals with varying cluster size *m* and number of clusters *n* as $\left(m,n\right)=\left\{\left(50,30\right),\left(25,60\right),\left(15,100\right)\right\}$. We simulated 2 continuous covariates following ${X}_{ij1}\sim N\left(0,4\right)$and ${X}_{ij2}\sim \mathrm{Unif}\left(-5,5\right)$, respectively. For the principal stratification model, we let $\boldsymbol\beta =\left\{-1,0.3,0.5\right\}$, and $\boldsymbol\gamma =\left\{-0.8,0.6,0.4\right\}$ so that the stratum proportions are 21.1% for never-survivors, 26.5% for protected individuals, and 52.4% for always-survivors. We generated the potential outcomes following Table 2, where we set ${\boldsymbol\alpha}_1^{11}=\left\{1.5,0.5,0.8\right\}$, ${\boldsymbol\alpha}_0^{11}=\left\{0.2,0.3,0.6\right\}$, and ${\boldsymbol\alpha}_1^{10}=\left\{-1.5,0.9,0.5\right\}$. We set ${\sigma}^2=5$and ${\tau}^2=1$ with an induced ICC of 0.167, which falls within the commonly reported range of 0–0.2 (32, 33). For each simulated data set under each combination of (*m*, *n*), we implemented the Bayesian sampler with 10,000 MCMC iterations (with the first 2,500 iterations as a burn-in). In addition, to evaluate the impact of varying cluster sizes, we considered scenarios with mean cluster sizes and numbers of clusters of $\left(\overline{m},n\right)=\left\{\left(50,30\right),\left(25,60\right),\left(15,100\right)\right\}$ and generated the data with a large coefficient of variability (CV) (defined as $\mathrm{CV}=\overline{m}/\sqrt{\mathrm{Var}(m)}$) of 1.0. Additional simulations with smaller outcome ICCs (0.01 or 0.05), smaller numbers of clusters and/or cluster sizes, and principal strata models including random intercepts (with an induced ICC of 0.05 or 0.10 on the latent response scale (34)) were also performed. We calculated the average posterior means, relative bias, and coverage across 200 simulated data sets. All analyses were performed using R software, version 4.0.1 (R Foundation for Statistical Computing, Vienna, Austria). R code for the simulation, including the data-generating process and the MCMC sampler, is available online.

### Analysis of the motivating trial example

We applied our methodology to analyze data from the Whole Systems Demonstrator (WSD) Telecare Questionnaire Study (35, 36). The WSD Telecare Study was a CRT randomized at the general practice level that evaluated the effect of telecare on the health-related quality of life and psychological well-being of 1,189 elderly recipients of social care in the United Kingdom over 12 months from 2008 to 2009. A total of 639 participants were randomized at the cluster level into the telecare arm, and 550 were randomized to usual care. Recipients were additionally clustered within general practices across 3 English local authorities. The rationale for the telecare intervention was not only its potential health benefit but also its advantage in terms of cost-effectiveness (37). There were 204 general practices in the study, and the cluster size varied from 1 to 26. The telecare arm installed electronic sensors in the homes of recipients that provided safety monitoring (e.g., recipient falls, fires in the home). Persons in the usual-care arm did not receive telecare. Our illustration focuses on health-related quality of life as measured via the EuroQol Group’s EQ-5D-VAS index, a self-rated scale (score range, 0–100) with 5 domains (mobility, self-care, usual activities, pain and discomfort, and anxiety and depression) measured at 12 months postrandomization (38, 39). Higher scores represent a better overall quality of life. The results reported herein vary slightly from the original published analysis due to different analytical methods and outcome data.

We considered trial participants to be nonsurvivors if they were deceased or had seriously deteriorated health, such that a self-reported health outcome for them could not be measured or collected and was thus undefined. Recipients with seriously deteriorated health included those who were too ill; were unable to continue due to dementia or deteriorated mental capacity; had moved to long-term nursing care, residential care, or sheltered housing; or had a family caregiver. For missing data on the baseline covariates and outcomes not due to death or seriously deteriorated health, we used multiple imputation (6, 13) to impute a single data set to fill those missing entries. Note that a fully Bayesian approach that incorporates the imputation in the proposed algorithm can also be implemented. Since the goal of our illustration was not focused on the missing-covariate problem, we did not pursue this direction. The resulting data set had 127 (10.7%) cases with truncated outcomes.

For both the principal stratification model and the potential outcome models, our baseline covariates included sex, age, ethnicity, participants’ highest level of education, an indicator for living in an only-adult household, number of comorbid conditions, impairment score, physical health score, mental health score, and EQ-5D-VAS index score (35). We used LMMs for the outcome regression model and multinomial logistic regression for the principal strata model, as previously specified. We did not pursue the more complex model that accounted for the random effects in the principal strata model because the average cluster size was too small and a handful of clusters had a size of 1, which would cause convergence issues (we discuss this further in the Discussion). Two MCMC chains of 100,000 iterations were implemented where the first 25,000 iterations were set as a burn-in. We started each chain using random initials. Model convergence and chain mixing were checked by means of trace plots. All analyses were performed using R 4.0.1. In addition, as a comparison model that might be frequently used in practice in the absence of our method, we fitted an LMM based on complete outcomes adjusting for the same baseline covariates.

## RESULTS

### Simulation study

Table 3 presents the simulation results for the key model parameters in the scenario of $\left(m,n\right)=\left(15,100\right)$. (Full results are shown in Web Appendix 2.) Relative bias and coverage are presented for ${\boldsymbol\alpha}_1^{11}$, ${\boldsymbol\alpha}_0^{11}$, the SACE, and the ICC. Our results show that the posterior mean values for most parameters were accurate, with less than 10% bias and more than 90% coverage. In particular, the SACE estimate was close to the truth (% bias = −5.2%), and the coverage probability was 0.93. The results for the other two scenarios of $\left(m,n\right)=\left\{\left(50,30\right),\left(25,60\right)\right\}$ were similar, where SACE estimates both had less than 5.0% bias and at least 0.95 coverage (see Web Tables 1–3 for full results). Additional simulations with variable cluster sizes (Web Tables 4–6), principal strata model specification with random effects (Web Tables 7–10), a small number of clusters and/or cluster sizes (Web Tables 11 and 12), and variable outcome ICCs (Web Tables 13 and 14) showed similar performance.

**Table 3:** Bias in Posterior Mean Values and Coverage for ${\boldsymbol \alpha}_\textsf{1}^{\textsf{11}}$, ${\boldsymbol \alpha}_\textsf{0}^{\textsf{11}}$, the Survivor Average Causal Effect, and the Intracluster Correlation Coefficient in a Scenario of (*m,n*)= ($\textsf{15,100}$)^a^

**Parameter and** **True Value**	**Posterior Mean**	**% Bias**	**Coverage**
$${\boldsymbol \alpha}_1^{11}=\left(\begin{array}{c}1.5\\{}0.5\\{}0.8\end{array}\right)$$	1.28	−14.4	0.93
	0.46	−7.5	0.95
	0.77	−3.8	0.95
$${\boldsymbol \alpha}_0^{11}=\left(\begin{array}{c}-1.5\\{}0.9\\{}0.5\end{array}\right)$$	−1.50	0.1	0.99
	0.90	−0.1	0.97
	0.50	−0.1	0.96
ICC = 0.17	0.17	0.0	0.96
SACE = 2.85	2.70	−5.2	0.93

Abbreviations: ICC, intracluster correlation coefficient; SACE, survivor average causal effect.

^a^ The results were based on 200 Markov chain Monte Carlo sampler simulations, each with 10,000 iterations and a burn-in of 2,500. Full results are provided in Web Appendix 2.

### Illustrative analysis

As noted above, our approach allows for adjustment for covariates and clustering. As is common in CRTs, several prognostic variables were preselected for adjustment in the primary analysis; the variables we used in our analysis are listed in Table 4. Our model identified 88.8% of recipients as always-survivors, 2.2% as protected individuals, and 8.9% as never-survivors. The posterior mean of the ICC was 0.003, with a 95% credible interval (i.e., 2.5% and 97.5% of the posterior sample) of (0.000, 0.020), suggesting small intracluster correlation in EQ-5D-VAS scores.

**Table 4:** Baseline Characteristics of 1,189 Participants in the Whole Systems Demonstrator Telecare Questionnaire Study, United Kingdom, 2008–2009^a^

**Covariate**	**Intervention**		**Control**		**Total**	
	**No.**	**%**	**No.**	**%**	**No.**	**%**
Sex						
Female	205	37.3	219	34.3	424	35.7
Male	345	62.7	420	65.7	765	64.3
Age, years^b^	73.9 (14.3)		74.3 (13.6)		74.1 (13.9)	
Ethnicity						
White	485	88.2	568	88.9	1,053	88.6
Non-white	65	11.8	71	11.1	136	11.4
Highest level of education						
No formal education	359	65.3	421	65.9	780	65.6
GCSE/O-levels	92	16.7	132	20.7	224	18.8
A-levels/HNC	29	5.3	42	6.6	71	6.0
University level	26	4.7	16	2.5	42	3.5
Graduate or professional	44	8.0	28	4.4	72	6.1
Adult-only household						
Yes	286	52.0	344	53.8	630	53.0
No	264	48.0	295	46.2	559	47.0
No. of comorbid conditions^b^	1.1 (1.5)		1.1 (1.4)		1.1 (1.5)	
Impairment score^b^	27.7 (14.3)		28.6 (15.6)		28.2 (15.1)	
Physical health score^b^	28.3 (8.7)		27.9 (8.5)		28.1 (8.6)	
Mental health score^b^	33.0 (8.0)		33.1 (7.8)		33.1 (7.9)	
EQ-5D-VAS index score^b^	52.7 (22.0)		53.2 (22.0)		53.0 (22.0)	

Abbreviations: GCSE, General Certificate of Secondary Education; HNC, Higher National Certificate.

^a^ Due to the use of a different imputation method, numbers vary slightly from those in the original trial publications (35, 36).

^b^ Values are expressed as mean (standard deviation).

Table 5 shows the analytical results of our analysis. The SACE point estimate for the effect of telecare on EQ-5D-VAS score was −0.70, with a 95% credible interval spanning potential effects that ranged from a decrease of −3.11 to an increase of 0.83. The estimated effect suggests that telecare did not markedly improve EQ-5D-VAS score in comparison with usual care in the principal stratum of always-survivors.

Web Figure 1 depicts the average number of always-survivors, protected individuals, and never-survivors by cluster based on the posterior sample of principal strata membership after burn-in. Many clusters had recipients that were possibly from all 3 strata. A notable advantage of our Bayesian approach is that the baseline characteristics of always-survivors can be obtained by averaging over the baseline covariates among always-survivors in the posterior sample of principal strata membership (summary provided in Web Table 15). In our illustration, demographic characteristics, socioeconomic status, and baseline health status among the always-survivors were similar to those in the overall population. This is unsurprising in this specific illustration, since 88.8% of recipients were identified as always-survivors.

Finally, in Table 5, the LMM point estimate based on the recipients with observed outcomes was −0.93 (95% confidence interval: −3.24, 1.38). This result was in line (again due to overlap in this specific illustration, but this is not guaranteed) with the SACE. However, it is important to note that while similar in our illustration, the LMM estimate does not have a causal interpretation because the analytical sample for the LMM estimate included recipients who were deemed to belong to the protected stratum. Intuitively, it suggests that the protected individuals in the telecare arm who were likely to die tended to be those with worse health outcomes in the treatment group.

**Table 5:** Results for the Survivor Average Causal Effect Estimate and the Proportion of Recipients in Each Principal Stratum With the Bayesian Joint Modeling and Linear Mixed-Effects Model Estimates

**Posterior**	**Point Estimate**	**95% CrI or 95% CI**
SACE	−0.70	−3.11, 0.83
$\overline{Y}(1)$	53.04	51.33, 54.74
$\overline{Y}(0)$	53.74	51.88, 55.03
Proportion of never-survivors	0.09	0.07, 0.11
Proportion of protected	0.02	0.00, 0.05
Proportion of always-survivors	0.89	0.88, 0.89
ICC	0.003	0.000, 0.020
Linear mixed-effects model	−0.93	−3.24, 1.38

Abbreviations: CI, confidence interval; CrI, credible interval; ICC, intracluster correlation coefficient; SACE, survivor average causal effect.

## DISCUSSION

### Conclusion

The SACE is a well-defined causal estimand that describes the effect of an intervention among participants who would survive regardless of their randomized assignment in a trial. We used Bayesian principal stratification to estimate the SACE in a CRT where the hierarchical data structure due to clustered randomization was accounted for in the modeling and data analysis. Since stratum membership is not fully identifiable for some participants, Bayesian estimation is a particularly attractive strategy with which to address uncertain stratum membership of the counterfactual survivor status. Notably, our approach considers the model-based credible interval estimation under the Bayesian framework, and therefore differs from the usual cluster-robust variance approach under the frequentist framework (40). The extent to which a cluster-robust variance approach applies to our Bayesian modeling framework merits additional research.

In our simulations, we observed low bias and good coverage of the true SACE parameter with our methods. In our analysis of the WSD telecare trial, 88.8% of the participants were identified as always-survivors, and the SACE suggested no significant change in the health-related quality of life measure (EQ-5D-VAS score) at 12 months (SACE = −0.70, 95% credible interval: −3.11, 0.83). This point estimate was slightly smaller than that from a complete-case analysis using a naive LMM model (point estimate = −0.93, 95% confidence interval: −3.24, 1.38). We note that our result is based on only a one-time measurement taken at 12 months, which differs from the original result published by Hirani et al. (35), where they utilized repeated outcome measures at different time points and considered different covariates. Adapting the principal stratification framework for CRTs with repeatedly measured outcomes requires additional methodological development. While our analysis of the WSD Telecare Study showed a limited effect on the EQ-5D-VAS outcome, telecare may affect other physical health outcomes or have cost-effectiveness properties due to prevention or earlier intervention on health needs.

### Practical recommendations

During the development and implementation of our methodology, we identified some analytical considerations that users may need to consider. First, the monotonicity assumption may be plausible for practice-level interventions like the one used in the WSD study setting, where installing electronic sensors in the telecare arm was unlikely to harm participants. Before the implementation of many medical trials, interventions are evaluated in pilot studies with safety monitoring; thus, this assumption may often be reasonable. Relaxing the monotonicity assumption by adding the “harmed stratum” (i.e., participants who die in the treatment group but survive in the control group) is possible. However, fitting the mixture model with an additional harmed stratum is an added layer of computational considerations, and additional simulations are needed to fully understand the benefit of including this stratum when the harmed population is relatively rare. Beyond the mixture modeling framework, other types of structural assumptions or sensitivity parameters are necessary to relax the monotonicity assumption (28, 29, 41, 42), and they represent a fruitful direction for future investigations in the context of CRTs.

Second, our simulation studies showed that the use of noninformative priors can achieve adequate performance without sufficient knowledge of key model parameters from existing studies. However, Bayesian approaches have an inherent advantage of leveraging existing knowledge through informative priors on key parameters to sharpen the model performance. For example, Turner et al. (43, 44) have demonstrated that compared with the noninformative priors, incorporating informative half-normal and β priors on the outcome ICC parameter (based on published ICC estimates) can produce narrower credible intervals for the outcome ICC and variance components parameters. We anticipate this finding to be applicable for estimating the SACE.

Third, while we have provided methods to account for clustering in the principal strata membership model, the model-fitting can be substantially more challenging than its counterpart without clustering. In the analysis of the WSD trial, the principal strata membership model failed to converge due to several extremely small clusters. Thus, we did not consider clustering in the principal strata membership model. Therefore, in practice, specifying more complex principal strata membership models often requires the absence of extremely small clusters. On the other hand, our additional simulation results (Web Tables 7–10) showed that when the ICC in the principal strata model is not exceeding 0.10, specifying the principal strata model without a random intercept can still achieve adequate performance characteristics and can be sufficient. However, we acknowledge that our assessment was limited to the data-generating process we considered, and a more systematic comparison between clustered and unclustered principal strata models in CRTs is warranted.

Lastly, our additional simulation (Web Tables 11 and 12) showed that our method can be employed to estimate the SACE in relatively small CRTs (e.g., 20 clusters with a cluster size of 25). We caution against using our model in even smaller CRTs, as additional research to address small-sample challenges in these settings is needed.

The use of the SACE in CRTs (and individually randomized trials) also requires some practical considerations from the design perspective, since the always-survivors stratum is a subset of trial participants that cannot be identified until after randomization and trial completion. Though it is a more attractive target estimand and thus alternative to composite outcomes, imputation, or other approaches to dealing with truncated outcomes, there is always a threat of loss of statistical power with the SACE because of the inherently smaller sample size of this stratum. However, this concern may be partially offset when the treatment effect is likely to be larger in the always-survivors stratum than in the overall population (i.e., average treatment effect) (45, 46). Relatedly, without knowing the size or characteristics of the always-survivors stratum prior to a study, power calculations may be a challenge, particularly when sample sizes are constrained and cannot be increased. Thus, we believe we can offer 3 practical recommendations for using the SACE in a trial. First, the SACE may be most ideal as a preplanned secondary analysis in trials with smaller available sample sizes, and only considered for the primary analysis in larger pragmatic trials or in trials where effect sizes and always-survivor rates can be anticipated with reasonable certainty to be in some range, and thus available sample sizes are adequate (45). Second, as is recommended when working with other uncertain trial design elements, we recommend that Monte Carlo simulation studies be undertaken to assess statistical power (47, 48). Jo (45) provides statistical power guidance for trials with treatment noncompliance that is potentially relevant to those using the SACE. Closed-form sample size solutions for noncompliance in CRTs could be extended to the SACE in future work. Third, when there is interest in conducting primary analysis to estimate the SACE, approaches for sample size reestimation with preplanned interim analysis (49) may be considered, but they may require future development.

## Supplementary Material

Web_Material_kwad038

## A. APPENDIX (Appendix follows)

### APPENDIX

The pseudoalgorithm for joint modeling of outcome and principal strata membership follows.

1. *Input:* Set multivariate normal priors for regression coefficients, $\boldsymbol{\alpha}_1^{11}$, $\boldsymbol{\alpha}_0^{11}$, $\boldsymbol{\alpha}_0^{10}$, $\boldsymbol\beta$, and $\boldsymbol\gamma$, and inverse **γ** priors for ${\sigma}^2$, ${\tau}^2$ (and ${\phi}^2$).
2. Set random initials for all parameters.
3. *For* S *iterations, do the following:*

1. Sample $\boldsymbol{\alpha}_1^{11}$, $\boldsymbol{\alpha}_0^{11}$, and $\boldsymbol{\alpha}_0^{10}$ from multivariate normal posteriors.
2. For each cluster, sample ${\eta}_i$ from a normal posterior.
3. Sample ${\tau}^2$ from an inverse γ posterior.
4. Sample ${\sigma}^2$ from an inverse γ posterior.
5. Sample $\boldsymbol\beta$ and $\boldsymbol\gamma$ jointly as follows:
  - Draw candidates of $\boldsymbol\beta$ and $\boldsymbol\gamma$ from a proposal distribution (mv-t dist.).
  - Compute the rejection ratio, α, based on likelihood and prior information.
  - Sample a random number $\boldsymbol\kappa$ from a Bernoulli(α) distribution.
  - If $\kappa =1$, then accept the candidates; if $\kappa =0$, then reject the candidates.
6. ^*^For each cluster, sample ${\chi}_i$ using the rejection sampling approach.
7. ^*^Sample ${\phi}^2$ from an inverse γ posterior.
8. Update the principal strata membership ${G}_{ij}$ following the Bayes rule.
9. Estimate the survivor average causal effect (SACE) among always-survivors (${G}_{ij}=11$).

1. *Output:* SACE.

## References

[1] Testa MA , Simonson DC. Assessment of quality-of-life outcomes. *N Engl J Med*. 1996;334(13):835–840. 8596551 10.1056/NEJM199603283341306

[2] Fairclough DL . Design and Analysis of Quality of Life Studies in Clinical Trials. New York, NY: Chapman & Hall, Inc.; 2010.

[3] Fayers PM , Hays R, Hays RD. Assessing Quality of Life in Clinical Trials: Methods and Practice. New York, NY: Oxford University Press; 2005.

[4] Rubin DB . Causal inference through potential outcomes and principal stratification: application to studies with “censoring” due to death. *Stat Sci*. 2006;21(3):299–309.

[5] Colantuoni E , Scharfstein DO, Wang C, et al. Statistical methods to compare functional outcomes in randomized controlled trials with high mortality. *BMJ*. 2018;360:j5748. 29298779 10.1136/bmj.j5748 PMC5751848

[6] Tong G , Li F, Allen AS. Missing data. In: Piantadosi S, Meinert C, eds. *Principles and Practice of Clinical Trials*. New York, NY: Springer Publishing Company; 2020:1–21.

[7] Carreras G , Miccinesi G, Wilcock A, et al. Missing not at random in end of life care studies: multiple imputation and sensitivity analysis on data from the ACTION Study. *BMC Med Res Methodol*. 2021;21(1):1–12. 33422019 10.1186/s12874-020-01180-y PMC7796568

[8] Fielding S , Fayers PM, McDonald A, et al. Simple imputation methods were inadequate for missing not at random (MNAR) quality of life data. *Health Qual Life Outcomes*. 2008;6(1):1–9. 18680574 10.1186/1477-7525-6-57 PMC2531086

[9] Harhay MO , Ratcliffe SJ, Small DS, et al. Measuring and analyzing length of stay in critical care trials. *Med Care*. 2019;57(9):e53–e59. 30664613 10.1097/MLR.0000000000001059 PMC6635104

[10] Lin W , Halpern SD, Prasad Kerlin M, et al. A “placement of death” approach for studies of treatment effects on ICU length of stay. *Stat Methods Med Res*. 2017;26(1):292–311. 25085115 10.1177/0962280214545121

[11] Neyman J . On the two different aspects of the representative method: the method of stratified sampling and the method of purposive selection. *J R Stat Soc*. 1934;97(4):558–625.

[12] Rubin DB . Estimating causal effects of treatments in randomized and nonrandomized studies. *J Educ Psychol*. 1974;66(5):688–701.

[13] Rubin DB . Multiple imputations in sample surveys—a phenomenological Bayesian approach to nonresponse. In: *Proceedings of the Section on Survey Research Methods: Papers Presented at the Annual Meeting of the American Statistical Association*. Vol. 1. Alexandria, VA: American Statistical Association; 1978:20–34.

[14] Suzuki E . Generalized causal measure: the beauty lies in its generality. *Epidemiology*. 2015;26(4):490–495. 25946224 10.1097/EDE.0000000000000304

[15] Imbens GW , Rubin DB. Bayesian inference for causal effects in randomized experiments with noncompliance. *Ann Stat*. 1997;25(1):305–327.

[16] Frangakis CE , Rubin DB, Zhou XH. Clustered encouragement designs with individual noncompliance: Bayesian inference with randomization, and application to advance directive forms. *Biostatistics*. 2002;3(2):147–164. 12933609 10.1093/biostatistics/3.2.147

[17] Zhang JL , Rubin DB, Mealli F. Likelihood-based analysis of causal effects of job-training programs using principal stratification. *J Am Stat Assoc*. 2009;104(485):166–176.

[18] Yang F , Small DS. Using post-outcome measurement information in censoring-by-death problems. *J R Stat Soc Ser B Stat Methodol*. 2016;78(1):299–318.

[19] McGuinness MB , Kasza J, Karahalios A, et al. A comparison of methods to estimate the survivor average causal effect in the presence of missing data: a simulation study. *BMC Med Res Methodol*. 2019;19(1):1–14. 31795945 10.1186/s12874-019-0874-x PMC6892197

[20] Forastiere L , Mealli F, VanderWeele TJ. Identification and estimation of causal mechanisms in clustered encouragement designs: disentangling bed nets using Bayesian principal stratification. *J Am Stat Assoc*. 2016;111(514):510–525. 28008210 10.1080/01621459.2015.1125788 PMC5166715

[21] Mattei A , Li F, Mealli F. Exploiting multiple outcomes in Bayesian principal stratification analysis with application to the evaluation of a job training program. *Ann Appl Stat*. 2013;7(4):2336–2360.

[22] Wang C , Scharfstein DO, Colantuoni E, et al. Inference in randomized trials with death and missingness. *Biometrics*. 2017;73(2):431–440. 27753071 10.1111/biom.12594 PMC6383567

[23] Rosenbaum PR . Comment: the place of death in the quality of life. *Stat Sci*. 2006;21(3):313–316.

[24] Murray DM , Taljaard M, Turner EL, et al. Essential ingredients and innovations in the design and analysis of group-randomized trials. *Annu Rev Public Health*. 2020;41:1–19. 31869281 10.1146/annurev-publhealth-040119-094027

[25] Turner EL , Li F, Gallis JA, et al. Review of recent methodological developments in group-randomized trials: part 1—design. *Am J Public Health*. 2017;107(6):907–915. 28426295 10.2105/AJPH.2017.303706 PMC5425852

[26] Zhang JL , Rubin DB. Estimation of causal effects via principal stratification when some outcomes are truncated by “death”. *J Educ Behav Stat*. 2003;28(4):353–368.

[27] Tong G , Seal KH, Becker WC, et al. Impact of complex, partially nested clustering in a three-arm individually randomized group treatment trial: a case study with the wHOPE Trial. *Clin Trials*. 2022;19(1):3–13. 34693748 10.1177/17407745211051288 PMC8847260

[28] Hayden D , Pauler DK, Schoenfeld D. An estimator for treatment comparisons among survivors in randomized trials. *Biometrics*. 2005;61(1):305–310. 15737107 10.1111/j.0006-341X.2005.030227.x

[29] Shepherd BE , Redman MW, Ankerst DP. Does finasteride affect the severity of prostate cancer? A causal sensitivity analysis. *J Am Stat Assoc*. 2008;103(484):1392–1404. 20526381 10.1198/016214508000000706 PMC2880822

[30] Neelon B , Gelfand AE, Miranda ML. A multivariate spatial mixture model for areal data: examining regional differences in standardized test scores. *J R Stat Soc Ser C Appl Stat*. 2014;63(5):737–761. 10.1111/rssc.12061 PMC4577245 26401059

[31] Haario H , Saksman E, Tamminen J. Componentwise adaptation for high dimensional MCMC. *Comput Stat*. 2005;20(2):265–273.

[32] Adams G , Gulliford MC, Ukoumunne OC, et al. Patterns of intra-cluster correlation from primary care research to inform study design and analysis. *J Clin Epidemiol*. 2004;57(8):785–794. 15485730 10.1016/j.jclinepi.2003.12.013

[33] Eldridge SM , Ashby D, Feder GS, et al. Lessons for cluster randomized trials in the twenty-first century: a systematic review of trials in primary care. *Clin Trials*. 2004;1(1):80–90. 16281464 10.1191/1740774504cn006rr

[34] Hedeker D . A mixed-effects multinomial logistic regression model. *Stat Med*. 2003;22(9):1433–1446. 12704607 10.1002/sim.1522

[35] Hirani SP , Beynon M, Cartwright M, et al. The effect of telecare on the quality of life and psychological well-being of elderly recipients of social care over a 12-month period: the Whole Systems Demonstrator cluster randomised trial. *Age Ageing*. 2014;43(3):334–341. 24333802 10.1093/ageing/aft185

[36] Henderson C , Knapp M, Fernández JL, et al. Cost effectiveness of telehealth for patients with long term conditions (Whole Systems Demonstrator telehealth questionnaire study): nested economic evaluation in a pragmatic, cluster randomised controlled trial. *BMJ*. 2013;346:f1035. 23520339 10.1136/bmj.f1035

[37] Bower P , Cartwright M, Hirani SP, et al. A comprehensive evaluation of the impact of telemonitoring in patients with long-term conditions and social care needs: protocol for the Whole Systems Demonstrator cluster randomised trial. *BMC Health Serv Res*. 2011;11(1):1–12. 21819569 10.1186/1472-6963-11-184 PMC3169462

[38] Ware JE Jr , Kosinski M, Turner-Bowker DM, et al. User’s Manual for the SF-12v2 Health Survey. 2nd ed. Lincoln, RI: QualityMetric Inc.; 2009.

[39] Feng Y , Parkin D, Devlin NJ. Assessing the performance of the EQ-VAS in the NHS PROMs programme. *Qual Life Res*. 2014;23(3):977–989. 24081873 10.1007/s11136-013-0537-z PMC4287662

[40] Liang KY , Zeger SL. Longitudinal data analysis using generalized linear models. *Biometrika*. 1986;73(1):13–22.

[41] Chiba Y , VanderWeele TJ. A simple method for principal strata effects when the outcome has been truncated due to death. *Am J Epidemiol*. 2011;173(7):745–751. 21354986 10.1093/aje/kwq418 PMC3070493

[42] Lou Y , Jones MP, Sun W. Estimation of causal effects in clinical endpoint bioequivalence studies in the presence of intercurrent events: noncompliance and missing data. *J Biopharm Stat*. 2019;29(1):151–173. 29995564 10.1080/10543406.2018.1489408

[43] Turner RM , Omar RZ, Thompson SG. Constructing intervals for the intracluster correlation coefficient using Bayesian modelling, and application in cluster randomized trials. *Stat Med*. 2006;25(9):1443–1456. 16220510 10.1002/sim.2304

[44] Turner RM , Omar RZ, Thompson SG. Bayesian methods of analysis for cluster randomized trials with binary outcome data. *Stat Med*. 2001;20(3):453–472. 11180313 10.1002/1097-0258(20010215)20:3<453::aid-sim803>3.0.co;2-l

[45] Jo B . Statistical power in randomized intervention studies with noncompliance. *Psychol Methods*. 2002;7(2):178–193. 12090409 10.1037/1082-989x.7.2.178

[46] Moerbeek M , van Schie S. What are the statistical implications of treatment non-compliance in cluster randomized trials: a simulation study. *Stat Med*. 2019;38(26):5071–5084. 31578760 10.1002/sim.8351 PMC6856967

[47] Landau S , Stahl D. Sample size and power calculations for medical studies by simulation when closed form expressions are not available. *Stat Methods Med Res*. 2013;22(3):324–345. 22491174 10.1177/0962280212439578

[48] Shi Y , Lee JH. Sample size calculations for group randomized trials with unequal group sizes through Monte Carlo simulations. *Stat Methods Med Res*. 2018;27(9):2569–2580. 30103663 10.1177/0962280216682775

[49] van Schie S , Moerbeek M. Re-estimating sample size in cluster randomised trials with active recruitment within clusters. *Stat Med*. 2014;33(19):3253–3268. 24719285 10.1002/sim.6172

