# Supplementary material for: A Bayesian Approach for Estimating the Survivor Average Causal Effect When Outcomes Are Truncated by Death in Cluster-Randomized Trials
Source: Am J Epidemiol. 2023 Feb 17;192(6):1006–15. doi: 10.1093/aje/kwad038 (PMC10236525; doi:10.1093/aje/kwad038)
Supplement: Web_Material_kwad038 [file web_material_kwad038.zip › kwad038 Tong Web Material.pdf]

WEB MATERIAL

for

A Bayesian Approach for Estimating the Survivor Average Causal Effect When Outcomes Are Truncated by Death in Cluster-Randomized Trials

Guangyu Tong*, Fan Li, Xinyuan Chen, Shashivadan P. Hirani, Stanton P. Newman, Wei Wang, and Michael O. Harhay

* Correspondence to Guangyu Tong, PhD, Department of Biostatistics, Yale School of Public Health, Room 234, 135 College Street, New Haven, CT 06510 (e-mail: guangyu.tong@yale.edu).

Table of Contents

WEB FIGURE 1. STACKED BAR PLOT FOR NUMBERS OF ALWAYS-SURVIVORS, PROTECTED, AND NEVER-SURVIVORS IDENTIFIED IN THE POSTERIOR SAMPLE 3

WEB APPENDIX 1. DETAILED DERIVATIONS FOR THE MARKOV CHAIN MONTE CARLO ALGORITHM 4

1.1 GIBBS SAMPLER FOR THE OUTCOME MODEL 4
1.2 METROPOLIS HASTING ALGORITHM FOR THE PRINCIPAL STRATA MODEL 7
1.3 GIBBS SAMPLER FOR THE PRINCIPAL STRATA MODEL USING A NESTED PROBIT SPECIFICATION 10

WEB APPENDIX 2. SIMULATIONS ASSESSING THE PERFORMANCE OF THE JOINT MODEL....... 14

WEB TABLE 1. POSTERIOR MEANS AND COVERAGES FOR SACE AND ALL MODEL PARAMETERS OVER 200 SIMULATIONS FOR $(m, n) = (50,30)$. 14
WEB TABLE 2. POSTERIOR MEANS AND COVERAGES FOR SACE AND ALL MODEL PARAMETERS OVER 200 SIMULATIONS FOR $(m, n) = (25,60)$. 15
WEB TABLE 3. POSTERIOR MEANS AND COVERAGES FOR SACE AND ALL MODEL PARAMETERS OVER 200 SIMULATIONS FOR $(m, n) = (15,100)$. 16
WEB TABLE 4. POSTERIOR MEANS AND COVERAGES FOR SACE AND ALL MODEL PARAMETERS OVER 200 SIMULATIONS FOR $(m, n) = (50,30)$ UNDER CV=1. 17
WEB TABLE 5. POSTERIOR MEANS AND COVERAGES FOR SACE AND ALL MODEL PARAMETERS OVER 200 SIMULATIONS FOR $(m, n) = (25,60)$ UNDER CV=1. 18
WEB TABLE 6. POSTERIOR MEANS AND COVERAGES FOR SACE AND ALL MODEL PARAMETERS OVER 200 SIMULATIONS FOR $(m, n) = (15,100)$ UNDER CV=1. 19
WEB TABLE 7. POSTERIOR MEANS AND COVERAGES FOR SACE AND ALL MODEL PARAMETERS OVER 200 SIMULATIONS FOR $(m, n) = (50,30)$. SIMULATION DATA GENERATED WITH RANDOM EFFECTS IN PRINCIPAL STRATA MODEL WITH $\phi 2 = 0.17$, INDUCING AN ICC OF 0.05. MODEL FITTED WITHOUT RANDOM EFFECTS IN THE PRINCIPAL STRATA MODEL. 20

WEB TABLE 8. POSTERIOR MEANS AND COVERAGES FOR SACE AND ALL MODEL PARAMETERS OVER 200 SIMULATIONS FOR $(m,n) = (50,30)$. SIMULATION DATA GENERATED WITH RANDOM EFFECTS IN PRINCIPAL STRATA MODEL WITH $\phi 2 = 0.17$, INDUCING AN ICC OF 0.05. MODEL FITTED WITH RANDOM EFFECTS IN PRINCIPAL STRATA MODEL. 21
WEB TABLE 9. POSTERIOR MEANS AND COVERAGES FOR SACE AND ALL MODEL PARAMETERS OVER 200 SIMULATIONS FOR $(m,n) = (50,30)$. SIMULATION DATA GENERATED WITH RANDOM EFFECTS IN PRINCIPAL STRATA MODEL WITH $\phi 2 = 0.4$, INDUCING AN ICC OF 0.10. MODEL FITTED WITHOUT RANDOM EFFECTS IN THE PRINCIPAL STRATA MODEL. 22
WEB TABLE 10. POSTERIOR MEANS AND COVERAGES FOR SACE AND ALL MODEL PARAMETERS OVER 200 SIMULATIONS FOR $(m,n) = (50,30)$. SIMULATION DATA GENERATED WITH RANDOM EFFECTS IN PRINCIPAL STRATA MODEL WITH $\phi 2 = 0.4$,. INDUCING AN ICC OF 0.10. MODEL FITTED WITH RANDOM EFFECTS IN THE PRINCIPAL STRATA MODEL. 23
WEB TABLE 11. POSTERIOR MEANS AND COVERAGES FOR SACE AND ALL MODEL PARAMETERS OVER 200 SIMULATIONS FOR $(m,n) = (75,20)$. 24
WEB TABLE 12. POSTERIOR MEANS AND COVERAGES FOR SACE AND ALL MODEL PARAMETERS OVER 200 SIMULATIONS FOR $(m,n) = (25, 20)$........ 25
WEB TABLE 13. POSTERIOR MEANS AND COVERAGES FOR SACE AND ALL MODEL PARAMETERS OVER 200 SIMULATIONS FOR $(m,n) = (30, 50)$. THE INDUCED OUTCOME ICC IS 0.05. 26
WEB TABLE 14. POSTERIOR MEANS AND COVERAGES FOR SACE AND ALL MODEL PARAMETERS OVER 200 SIMULATIONS FOR $(m,n) = (30, 50)$. THE INDUCED OUTCOME ICC IS 0.01. 27
WEB TABLE 15. DESCRIPTIVE STATISTICS FOR ALWAYS-SURVIVORS BY INTERVENTION ARMS BASED ON BAYESIAN POSTERIOR SAMPLE FOR PRINCIPAL STRATA MEMBERSHIP........ 28

Web Figure 1. Stacked bar plot for numbers of always-survivors, protected, and never-survivors identified in the posterior sample. The cluster sizes of the general practices vary from 1 to 26, and there are 204 clusters.

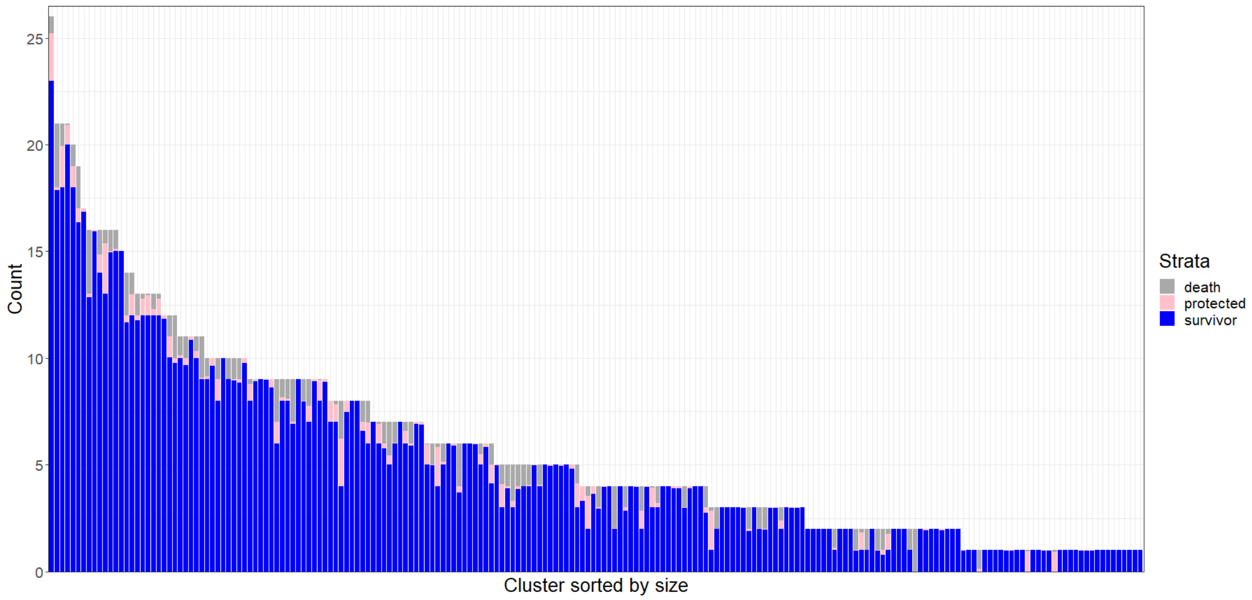

Web Appendix 1. Detailed derivations for the Markov Chain Monte Carlo algorithm

1.1 Gibbs sampler for the outcome model:

Step 1:

The prior distributions for the regression coefficients are multivariate normal:

$$\alpha_1^{11} \sim MVN(a_1^{11}, \Sigma_1^{11})$$

$$\alpha_0^{11} \sim MVN(a_0^{11}, \Sigma_0^{11})$$

$$\alpha_1^{10} \sim MVN(a_1^{10}, \Sigma_1^{10})$$

Diffuse priors such as $MVN(\mathbf{0}, diag(1000))$ can be specified in practice.

Priors for the variance parameters are inverse gamma:

$$\tau^2 \sim IG(a, b)$$

$$\sigma^2 \sim IG(c, d)$$

Diffuse priors such as $IG(shape = 0.001, rate = 0.001)$ can be specified here. Then, conditional on known strata information from the data augmentation, the posteriors can be derived as follows.

Step 2.1:

(1) For α_1^{11}:

$$\alpha_1^{11} \sim MVN(m_1^{11}, v_1^{11})$$

$$v_1^{11} = \left[\sigma^{-2} * \sum_{i=1}^{S} \sum_{j \in G_{11}} D_i * X_{ij} X_{ij}^T + (\Sigma_1^{11})^{-1}\right]^{-1}$$

$$m_1^{11} = v_1^{11} \left[\sigma^{-2} * \sum_{i=1}^{S} \sum_{j \in G_{11}} D_i * X_{ij}(y_{ij} - \eta_i) + (\Sigma_1^{11})^{-1} a_1^{11}\right]$$

(2) For α_0^{11}:

$$\alpha_0^{11} \sim MVN(m_0^{11}, v_0^{11})$$

$$v_0^{11} = \left[\sigma^{-2} * \sum_{i=1}^{s} \sum_{j \in G_{11}} (1 - D_i) * X_{ij} X_{ij}^T + (\Sigma_0^{11})^{-1}\right]^{-1}$$

$$m_0^{11} = v_0^{11}\left[\sigma^{-2} * \sum_{i=1}^{s} \sum_{j \in G_{11}} (1 - D_i) * X_{ij}\left(y_{ij} - \eta_i\right) + (\Sigma_0^{11})^{-1} a_0^{11}\right]$$

(3) For α_1^{10}:

$$\alpha_1^{10} \sim MVN(m_1^{10}, v_1^{10})$$

$$v_1^{10} = \left[\sigma^{-2} * \sum_{i=1}^{s} \sum_{j \in G_{10}} D_i * X_{ij} X_{ij}^T + (\Sigma_1^{10})^{-1}\right]^{-1}$$

$$m_1^{10} = v_1^{10}\left[\sigma^{-2} * \sum_{i=1}^{s} \sum_{j \in G_{10}} D_i * X_{ij}\left(y_{ij} - \eta_i\right) + (\Sigma_1^{10})^{-1} a_1^{10}\right]$$

Step 2.2:

Let $n_i = \sum_j S_{ij}$ be the number of individuals in ith cluster that are alive. For η_i,

$$\eta_i \sim N\left(m_{\eta_i}, v_{\eta_i}\right)$$

$$v_{\eta_i} = \left(\frac{n_i}{\sigma^2} + \frac{1}{\tau^2}\right)^{-1}$$

$$m_{\eta_i} = v_{\eta_i} \sigma^{-2} \sum_{i=1}^{s} D_i \left[\sum_{j \in G_{11}} \left(y_{ij} - X_{ij}^T \alpha_1^{11}\right) + \sum_{j \in G_{10}} \left(y_{ij} - X_{ij}^T \alpha_1^{10}\right)\right]$$

$$+ v_{\eta_i} \sigma^{-2} \sum_{i=1}^{s} (1 - D_i) \left[\sum_{j \in G_{11}} \left(y_{ij} - X_{ij}^T \alpha_0^{11}\right)\right]$$

Step 2.3:

For τ^2,

$$\tau^2 \sim IG\left(\text{shape} = a + \frac{s}{2}, \text{rate} = \ b + \frac{\sum_{i=1}^{s} \eta_i^2}{2}\right)$$

Step 2.4:

Let $N = \sum_{i=1}^{s} \sum_j S_{ij}$ be the total number of individuals alive. For σ^2,

$$\sigma^2 \sim IG(\text{shape} = c^*, \text{rate} = d^*)$$

$$c^* = c + \frac{N}{2}$$

$$d^* = d + \frac{1}{2}\sum_{i=1}^{s} D_i \left[\sum_{j \in G_{11}} \left(y_{ij} - X_{ij}^T \alpha_1^{11} - \eta_i\right)^2 + \sum_{j \in G_{10}} \left(y_{ij} - X_{ij}^T \alpha_1^{10} - \eta_i\right)^2\right]$$

$$+ \frac{1}{2}\sum_{i=1}^{s} (1 - D_i) \sum_{j \in G_{11}} \left(y_{ij} - X_{ij}^T \alpha_0^{11} - \eta_i\right)^2$$

1.2 Metropolis Hasting algorithm for the principal strata model:

We include the random effect term of χ_i in this derivation, which can be omitted when this term is not meaningful or the cluster sizes are small. We chose the always-survivors ($G_{ij} = 11$) as the reference group given it is normally the dominant strata. The likelihood for the principal strata model is,

$$\prod_{i=1}^{s}\prod_{j=1}^{n_i}\left[\frac{e^{X_{ij}^T\beta+\chi_i}}{1+e^{X_{ij}^T\beta+\chi_i}+e^{X_{ij}^T\gamma+\chi_i}}\right]^{I(G_{ij}=00)}\left[\frac{e^{X_{ij}^T\gamma+\chi_i}}{1+e^{X_{ij}^T\beta+\chi_i}+e^{X_{ij}^T\gamma+\chi_i}}\right]^{I(G_{ij}=10)}\left[\frac{1}{1+e^{X_{ij}^T\beta+\chi_i}+e^{X_{ij}^T\gamma+\chi_i}}\right]^{I(G_{ij}=11)}$$

We choose multivariate normal priors for β and γ and use the Metropolis Hasting algorithm for model estimation. We choose multivariate *t* distributions, $t_3(s_\beta T_\beta)$ and $t_3(s_\gamma T_\gamma)$ as the proposal distributions that center at the values of the previous iteration. The parameter s_β and s_γ scales the covariance to achieve optimal acceptance rates; $T_\beta$and $T_\gamma$each is a component-specific scale matrix. An adaptive proposal approach can be used to tune T_β and T_γ by utilizing empirical covariance from an extended burn-in. Similarly, χ_i' can also be updated using this approach. We specify the prior distributions for the regression coefficients as multivariate normal distributions:

$$\beta \sim MVN(\beta_0, \Lambda)$$

$$\gamma \sim MVN(\gamma_0, \Gamma)$$

Diffuse priors such as $MVN(\mathbf{0}, diag(1000))$ can be specified in practice. We also specify the priors for the random effects variance as the following inverse gamma distribution:

$$\phi^2 \sim IG(g, h)$$

Again, we can specific an inverse-gamma prior $IG(0.001{,}0.001)$ here. Below are the steps to obtain posteriors.

Step 2.5:

Update β and γ:

(1) Sample candidate z_β from multivariate t distribution, $t_3(s_\beta T_\beta)$, where s_β is tuning parameter and T_β is the covariance of β in previous steps. Here, define the number of burnin steps as b. Then $T_\beta = diag_3(1)$ for the first $2b$ steps, and T_β is the covariance of chain of β between the bth and $2b$th steps.

(2) Sample candidate z_γ with the same approach as (i).

(3) Denote β^t and γ^tas the value of β and γ in the tth step; $l(\cdot)$ is the multinomial likelihood. For step *t*, calculate

$$\alpha = min\left(1,\ \frac{f_0(z_\beta)f_0(z_\gamma)l(z_\beta)l(z_\gamma)\ t_3(\beta^t|z_\beta)t_3(\gamma^t|z_\gamma)}{f_0(\beta^t)f_0(\gamma^t)l(\beta^t)l(\gamma^t)\ t_3(z_\beta|\beta^t)t_3(z_\gamma|\gamma^t)}\right)$$

(4) Sample from $Bernoulli(\alpha)$; Accept z_β and z_γ if the sampled value is smaller than α. Otherwise, reject the candidate.

Step 2.6*:

Update χ_i if random effects for principal strata model is included: Define W as the design matrix for the random effects and follow the same above procedure to update χ_i.

Step 2.7*:

Sample the variance of random effects:

$$\phi^2 \sim IG\left(\text{shape} = g + s, \text{rate} = h + \tfrac{1}{2}\textstyle\sum_{i=1}^{s}\chi_i^2\right)$$

Step 2.8:

Update the principal strata membership under the following four conditions:

(1) If $D_i = 1\ and\ S_{ij}(1) = 0$, then $G_{ij} = 00$.

(2) If $D_i = 0\ and\ S_{ij}(0) = 1$, then $G_{ij} = 11$.

(3) If $D_i = 0, and\ S_{ij}(0) = 0$, then

$$P_{ij}(00) = \frac{e^{X_{ij}^T\beta+\chi_i}}{e^{X_{ij}^T\beta+\chi_i}+e^{X_{ij}^T\gamma+\chi_i}+1}$$

$$P_{ij}(10) = \frac{e^{X_{ij}^T\gamma+\chi_i'}}{e^{X_{ij}^T\beta+\chi_i}+e^{X_{ij}^T\gamma+\chi_i}+1}$$

$$M_{ij} \sim Bernoulli\left(\frac{P_{ij}(00)}{P_{ij}(00)+P_{ij}(10)}\right)$$

If $M_{ij} = 1$, $G_{ij} = 00$; If $M_{ij} = 0$, $G_{ij} = 10$.

(4) if $D_i = 1, and\ S_{ij}(1) = 1$, then

$$P_{ij}(10) = \frac{e^{X_{ij}^T\gamma+\chi_i}}{e^{X_{ij}^T\beta+\chi_i}+e^{X_{ij}^T\gamma+\chi_i}+1} * p_N\left(\frac{y_{ij}- X_{ij}^T\alpha_1^{10}+\eta_i}{\sigma}\right)$$

$$P_{ij}(11) = \frac{1}{e^{X_{ij}^T\beta+\chi_i}+e^{X_{ij}^T\gamma+\chi_i}+1} * p_N\left(\frac{y_{ij}- X_{ij}^T\alpha_1^{11}+\eta_i}{\sigma}\right)$$

$$M_{ij} \sim Bernoulli\left(\frac{P_{ij}(10)}{P_{ij}(10)+P_{ij}(11)}\right)$$

If $M_{ij} = 1$, $G_{ij} = 10$; If $M_{ij} = 0$, $G_{ij} = 11$.

In together with the outcome regression model, we can obtain the SACE in the final step conditional on the always-survivor strata.

1.3 Gibbs sampler for the principal strata model using a nested Probit specification:

We first define a nested Probit model for the strata membership. Under the monotonicity assumption, there are three strata for the nested Probit model, always-survivors: $G_{ij} = 11$ if $S_{ij}(1) = S_{ij}(0) = 1$; protected: $G_{ij} = 10$ if $S_{ij}(1) = 1$ but $S_{ij}(0) = 0$; never-survivors: $G_{ij} = 00$ if $S_{ij}(1) = S_{ij}(0) = 0$. Define the numbers of patients in these three strata as n_{11}, n_{10}, and n_{00}. The membership of strata is known for some of the patients including survivors in the control group and the deceased in the treatment group, but unknown for survivors in the treatment group or the deceased in the control group. The first layer of the model will be whether a patient is in ($G_{ij} = 11$), and the second layer of the model will be whether a patient is in ($G_{ij} = 10$). Define β and γ as the regression coefficients for the first and second layer. Define χ_i as the cluster-level random effects with mean zero and variance of ϕ^2; the proportion of observations in the never-survivors group ($G_{ij} = 00$) as θ. The nested Probit model then can be expressed as the follows,

$$P(G_{ij} = 00) = 1 - \Psi(X_{ij}^T\beta + \chi_i)$$

$$P(G_{ij} = 10) = \left(1 - P(G_{ij} = 00)\right) P\left(1 - \Psi(X_{ij}^T\gamma + \chi_i)\right) = (1 - \theta)\left(1 - \Psi(X_{ij}^T\gamma + \chi_i)\right)$$

The posteriors for the principal stratification model can be obtained with the following Gibbs sampler:

Step 1:

Set the prior distributions for the regression coefficients as multivariate normal:

$$\beta \sim MVN(\beta_0, \Lambda)$$

$$\gamma \sim MVN(\gamma_0, \Gamma)$$

Diffuse priors such as $MVN(\mathbf{0}, diag(1000))$ can be specified in practice.

Set the prior for the variance parameters are inverse gamma:

$$\phi^2 \sim IG(g, h)$$

Diffuse priors such as $IG(shape = 0.001, rate = 0.001)$ can be specified here.

Step 2.1:

For β,

$$\beta \sim MVN(m_\beta, v_\beta)$$

$$v_\beta = \left[\sum_{i=1}^{s}\sum_{j} X_{ij}X_{ij}^T + \Lambda^{-1}\right]^{-1}$$

$$m_\beta = v_\beta\left[\sum_{i=1}^{s}\sum_{j}(Z_{ij} - \chi_i)X_{ij} + \Lambda^{-1}\beta_0\right]$$

Step 2.2:

For γ,

$$\gamma \sim MVN(m_\gamma, v_\gamma)$$

$$v_\gamma = \left[\left(ij \in (G_{10}, G_{11})\right)\sum_{i=1}^{s}\sum_{j} X_{ij}X_{ij}^T + \Gamma^{-1}\right]^{-1}$$

$$m_\gamma = v_\gamma\left[(ij \in (G_{10}, G_{11}))\sum_{i=1}^{s}\sum_{j}(W_{ij} - \chi_i)X_{ij} + \Gamma^{-1}\gamma_0\right]$$

Step 2.3:

For ϕ^2,

$$\phi^2 \sim IG\left(\text{shape} = g + s, \text{rate} = h + \tfrac{1}{2}\textstyle\sum_{i=1}^{s}\chi_i^2\right)$$

Step 2.4:

Define n_{00i}, n_{10i}, and n_{11i} are the total numbers of patients in each stratum of each cluster, and the total sample size of each cluster is n_i^*. For χ_i,

$$\chi_i \sim N\left(m_{\chi_i}, v_{\chi_i}\right)$$

$$v_{\chi_i} = \left(n_{00i} + n_{10i} + n_{11i} + \frac{1}{\phi^2}\right)^{-1} = \left(n_i^* + \frac{1}{\phi^2}\right)^{-1}$$

$$m_{\chi_i} = v_{\chi_i}\left[\sum_{j=1}^{n_i^*}\left(Z_{ij} - X_{ij}^T\beta\right)\right]$$

Step 2.5:

Update the strata membership of each patient,

(a) if $D_i = 1$ and $S_{ij}(1) = 0$, then $G_{ij} = 00$.

(b) if $D_i = 0$ and $S_{ij}(0) = 1$, then $G_{ij} = 11$.

(c) if $D_i = 0, and$ $S_{ij}(0) = 0$, then

$$P_{ij}(00) = \Phi\left(X_{ij}^T\beta + \chi_i\right)$$

$$P_{ij}(10) = \left(1 - \Phi\left(X_{ij}^T\beta + \chi_i\right) * \Phi\left(X_{ij}^T\gamma + \chi_i\right)\right)$$

$$M_{ij} \sim Bernoulli\left(\frac{P_{ij}(00)}{P_{ij}(00) + P_{ij}(10)}\right)$$

If $M_{ij} = 1$, $G_{ij} = 00$; If $M_{ij} = 0$, $G_{ij} = 10$.

(d) if $D_i = 1, and$ $S_{ij}(1) = 1$, then

$$P_{ij}(10) = \Phi\left(X_{ij}^T\gamma + \chi_i\right) * p_N\left(\frac{Y_{ij} - X_{ij}^T\alpha_1^{10} - \eta_i}{\sigma}\right)$$

$$P_{ij}(11) = \left(1 - \Phi\left(X_{ij}^T\gamma + \chi_i\right)\right) * p_N\left(\frac{Y_{ij} - X_{ij}^T\alpha_1^{11} - \eta_i}{\sigma}\right)$$

$$M_{ij} \sim Bernoulli\left(\frac{P_{ij}(10)}{P_{ij}(10) + P_{ij}(11)}\right)$$

If $M_{ij} = 1$, $G_{ij} = 10$; If $M_{ij} = 0$, $G_{ij} = 11$.

Step 2.6:

For each Z_{ij}, sample from the following truncated normal distributions,

$$Z_{ij} \sim N_{Z_{ij}>0}\left(X_{ij}^T\beta + \chi_i, 1\right), if\ \left(G_{ij} = 00\right)$$

$$Z_{ij} \sim N_{Z_{ij}\leq 0}\left(X_{ij}^T\beta + \chi_i, 1\right), if\ \left(G_{ij} = 10|11\right)$$

Step 2.7:

For each $W_{ij\in(G_{10},G_{11})}$, sample from the following truncated normal distributions,

$$W_{ij\in(G_{10},G_{11})} \sim N_{W_{ij}>0}\left(X_{ij}^T\gamma + \chi_i, 1\right), if\ \left(G_{ij} = 10\right)$$

$$W_{ij\in(G_{10},G_{11})} \sim N_{W_{ij}\leq 0}\left(X_{ij}^T\gamma + \chi_i, 1\right), if\ \left(G_{ij} = 11\right)$$

$$W_{ij} = 0, if\ G_{ij} = 00$$

Step 2.8:

Update the values of n_{00}, n_{10} and n_{11} based on the results in the last step. The value of θ is $n_{00}/(n_{00} + n_{10} + n_{11})$.

Web Appendix 2. Simulations assessing the performance of the joint model.

Web Table 1. Posterior means and coverages for SACE and all model parameters over 200 simulations for $(m,n) = (50,30)$.

Parameter	True values	Posterior mean	%bias	Coverage
$\boldsymbol{\alpha_1^{11}}$	1.50	1.35	-9.93	0.95
	0.50	0.46	-7.89	0.95
	0.80	0.78	-3.12	0.93
$\boldsymbol{\alpha_1^{10}}$	0.20	0.59	192.50	0.96
	0.30	0.24	-21.42	0.95
	0.60	0.54	-9.54	0.93
$\boldsymbol{\alpha_0^{11}}$	-1.50	-1.46	-2.94	0.96
	0.90	0.90	0.29	0.94
	0.50	0.50	0.30	0.95
$\boldsymbol{\beta}$	-1.00	-1.02	2.46	0.67
	0.30	0.31	2.93	0.70
	0.50	0.51	1.77	0.67
$\boldsymbol{\gamma}$	-0.80	-0.82	2.25	0.69
	0.60	0.60	0.72	0.63
	0.40	0.41	1.94	0.69
τ^2	1.00	1.10	10.01	0.98
σ^2	5.00	4.97	-0.53	0.97
ICC	0.17	0.18	6.09	0.98
**SACE**	**2.85**	**2.71**	**-4.82**	**0.96**
$G = 00$	0.21	0.21	-0.78	0.79
$G = 10$	0.26	0.26	-0.09	0.82
$G = 11$	0.52	0.53	0.36	0.67

Web Table 2. Posterior means and coverages for SACE and all model parameters over 200 simulations for $(m, n) = (25,60)$.

Parameter	True values	Posterior mean	%bias	Coverage
$\boldsymbol{\alpha_1^{11}}$	1.50	1.27	-15.64	0.93
	0.50	0.46	-8.06	0.98
	0.80	0.77	-3.91	0.96
$\boldsymbol{\alpha_1^{10}}$	0.20	0.63	216.49	0.95
	0.30	0.22	-27.88	0.96
	0.60	0.55	-8.15	0.96
$\boldsymbol{\alpha_0^{11}}$	-1.50	-1.52	1.36	0.96
	0.90	0.91	1.39	0.96
	0.50	0.50	0.09	0.95
$\boldsymbol{\beta}$	-1.00	-1.01	1.05	0.63
	0.30	0.31	2.09	0.64
	0.50	0.51	1.51	0.62
$\boldsymbol{\gamma}$	-0.80	-0.80	0.54	0.62
	0.60	0.61	1.62	0.62
	0.40	0.41	1.87	0.63
τ^2	1.00	1.04	4.44	0.97
σ^2	5.00	5.00	0.03	0.98
ICC	0.17	0.17	2.41	0.97
**SACE**	**2.85**	**2.71**	**-4.89**	**0.95**
$G = 00$	0.21	0.21	-0.48	0.71
$G = 10$	0.26	0.27	0.34	0.77
$G = 11$	0.52	0.52	0.02	0.59

Web Table 3. Posterior means and coverages for SACE and all model parameters over 200 simulations for $(m, n) = (15{,}100)$.

Parameter	True values	Posterior mean	%bias	Coverage
$\boldsymbol{\alpha_1^{11}}$	1.50<br>0.50<br>0.80	1.28<br>0.46<br>0.77	-14.42<br>-7.45<br>-3.83	0.93<br>0.95<br>0.95
$\boldsymbol{\alpha_1^{10}}$	0.20<br>0.30<br>0.60	0.65<br>0.22<br>0.54	223.17<br>-26.42<br>-10.01	0.91<br>0.94<br>0.96
$\boldsymbol{\alpha_0^{11}}$	-1.50<br>0.90<br>0.50	-1.50<br>0.90<br>0.50	0.13<br>-0.11<br>-0.10	0.99<br>0.97<br>0.96
$\boldsymbol{\beta}$	-1.00<br>0.30<br>0.50	-1.01<br>0.30<br>0.51	0.92<br>0.07<br>1.22	0.61<br>0.62<br>0.65
$\boldsymbol{\gamma}$	-0.80<br>0.60<br>0.40	-0.80<br>0.62<br>0.40	0.32<br>2.96<br>0.32	0.60<br>0.62<br>0.62
τ^2	1.00	1.01	0.74	0.95
σ^2	5.00	4.98	-0.33	0.96
ICC	0.17	0.17	0.01	0.96
**SACE**	**2.85**	**2.70**	**-5.24**	**0.93**
$G = 00$<br>$G = 10$<br>$G = 11$	0.21<br>0.26<br>0.52	0.21<br>0.27<br>0.52	-0.89<br>0.67<br>0.02	0.80<br>0.79<br>0.66

Web Table 4. Posterior means and coverages for SACE and all model parameters over 200 simulations for $(\bar{m}, n) = (50{,}30)$ under CV=1.

Parameter	True values	Posterior mean	%bias	Coverage
$\boldsymbol{\alpha_1^{11}}$	1.50	1.25	-16.78	0.93
	0.50	0.45	-9.00	0.97
	0.80	0.76	-4.93	0.92
$\boldsymbol{\alpha_1^{10}}$	0.20	0.54	168.56	0.98
	0.30	0.23	-23.16	0.96
	0.60	0.56	-6.92	0.98
$\boldsymbol{\alpha_0^{11}}$	-1.50	-1.44	-3.71	0.95
	0.90	0.89	-0.67	0.95
	0.50	0.50	-0.71	0.96
$\boldsymbol{\beta}$	-1.00	-1.02	1.64	0.71
	0.30	0.30	0.13	0.77
	0.50	0.51	1.29	0.73
$\boldsymbol{\gamma}$	-0.80	-0.81	0.97	0.71
	0.60	0.60	0.59	0.67
	0.40	0.40	1.14	0.67
τ^2	1.00	1.08	8.34	0.96
σ^2	5.00	4.98	-0.46	0.95
ICC	0.17	0.17	4.03	0.96
**SACE**	**2.85**	**2.61**	**-8.28**	**0.95**
$G = 00$	0.21	0.21	-0.45	0.74
$G = 10$	0.26	0.27	0.32	0.81
$G = 11$	0.52	0.52	0.02	0.67

Note: Variable cluster sizes were generated with Gamma(α, β) distribution with the shape parameter $\alpha = 1/CV^2$ and rate parameter $\beta = 1/(\bar{m}CV^2)$. Generated values were rounded to integers.

Web Table 5. Posterior means and coverages for SACE and all model parameters over 200 simulations for $(\bar{m}, n) = (25{,}60)$ under CV=1.

Parameter	True values	Posterior mean	%bias	Coverage
$\boldsymbol{\alpha_1^{11}}$	1.50<br>0.50<br>0.80	1.27<br>0.45<br>0.76	-15.35<br>-10.04<br>-4.49	0.90<br>0.92<br>0.95
$\boldsymbol{\alpha_1^{10}}$	0.20<br>0.30<br>0.60	0.59<br>0.24<br>0.56	195.85<br>-21.45<br>-7.25	0.94<br>0.91<br>0.96
$\boldsymbol{\alpha_0^{11}}$	-1.50<br>0.90<br>0.50	-1.53<br>0.90<br>0.49	2.23<br>0.09<br>-1.30	0.95<br>0.94<br>0.94
$\boldsymbol{\beta}$	-1.00<br>0.30<br>0.50	-1.01<br>0.30<br>0.51	0.54<br>-0.06<br>1.45	0.63<br>0.62<br>0.66
$\boldsymbol{\gamma}$	-0.80<br>0.60<br>0.40	-0.81<br>0.61<br>0.40	1.23<br>1.11<br>1.24	0.60<br>0.62<br>0.61
τ^2	1.00	1.06	5.68	0.95
σ^2	5.00	4.97	-0.51	0.97
ICC	0.17	0.17	3.32	0.95
**SACE**	**2.85**	**2.72**	**-4.59**	**0.97**
$G = 00$<br>$G = 10$<br>$G = 11$	0.21<br>0.26<br>0.52	0.21<br>0.27<br>0.52	0.20<br>0.34<br>-0.26	0.74<br>0.81<br>0.69

Note: Variable cluster sizes were generated with Gamma(α, β) distribution with the shape parameter $\alpha = 1/CV^2$, and rate parameter $\beta = 1/(\bar{m}CV^2)$. Generated values were rounded to integers.

Web Table 6. Posterior means and coverages for SACE and all model parameters over 200 simulations for $(\bar{m}, n) = (15{,}100)$ under CV=1.

Parameter	True values	Posterior mean	%bias	Coverage
$\boldsymbol{\alpha_1^{11}}$	1.50	1.26	-16.20	0.88
	0.50	0.45	-10.72	0.93
	0.80	0.77	-3.73	0.93
$\boldsymbol{\alpha_1^{10}}$	0.20	0.65	222.88	0.94
	0.30	0.22	-27.13	0.96
	0.60	0.55	-8.59	0.96
$\boldsymbol{\alpha_0^{11}}$	-1.50	-1.47	-1.92	0.97
	0.90	0.90	0.37	0.99
	0.50	0.50	0.37	0.94
$\boldsymbol{\beta}$	-1.00	-1.00	0.09	0.66
	0.30	0.30	0.47	0.67
	0.50	0.50	0.54	0.69
$\boldsymbol{\gamma}$	-0.80	-0.83	3.67	0.65
	0.60	0.61	0.90	0.67
	0.40	0.41	1.80	0.67
τ^2	1.00	0.99	-1.38	0.91
σ^2	5.00	5.00	-0.06	0.95
ICC	0.17	0.16	-0.02	0.92
**SACE**	**2.85**	**2.65**	**-6.97**	**0.89**
$G = 00$	0.21	0.21	0.38	0.72
$G = 10$	0.26	0.26	-0.88	0.82
$G = 11$	0.52	0.53	0.29	0.70

Note: Variable cluster sizes were generated with Gamma(α, β) distribution with the shape parameter $\alpha = 1/CV^2$ and rate parameter $\beta = 1/(\bar{m}CV^2)$. Generated values were rounded to integers.

Web Table 7. Posterior means and coverages for SACE and all model parameters over 200 simulations for $(m, n) = (50{,}30)$. Simulation data generated with random effects in principal strata model with $\phi^2 = 0.17$, inducing an ICC of 0.05. Model fitted *without* random effects in the principal strata model.

**Parameter**	**True values**	**Posterior mean**	**%bias**	**Coverage**
$\boldsymbol{\alpha_1^{11}}$	1.50	1.20	-19.74	0.95
	0.50	0.40	-19.08	0.92
	0.80	0.69	-13.97	0.95
$\boldsymbol{\alpha_1^{10}}$	0.20	0.84	317.85	0.92
	0.30	0.17	-42.15	0.94
	0.60	0.58	-3.83	0.93
$\boldsymbol{\alpha_0^{11}}$	-1.50	-1.53	2.07	0.95
	0.90	0.89	-0.71	0.96
	0.50	0.51	1.96	0.95
$\boldsymbol{\beta}$	-1.00	-1.00	-0.39	0.85
	0.30	0.29	-3.38	0.93
	0.50	0.49	-2.54	0.93
$\boldsymbol{\gamma}$	-0.80	-0.85	6.49	0.73
	0.60	0.61	1.50	0.91
	0.40	0.38	-4.88	0.86
τ^2	1.00	1.14	13.54	0.96
σ^2	5.00	5.00	-0.07	0.96
ICC	0.17	0.18	8.53	0.95
**SACE**	**3.37**	**3.14**	**-6.89**	**0.95**
$G = 00$	0.18	0.20	1.13	0.73
$G = 10$	0.26	0.29	-1.53	0.65
$G = 11$	0.45	0.51	0.43	0.52

Web Table 8. Posterior means and coverages for SACE and all model parameters over 200 simulations for $(m, n) = (50{,}30)$. Simulation data generated with random effects in principal strata model with $\phi^2 = 0.17$, inducing an ICC of 0.05. Model fitted with random effects in principal strata model.

**Parameter**	**True values**	**Posterior mean**	**%bias**	**Coverage**
$\boldsymbol{\alpha_1^{11}}$	1.50	1.20	-20.22	0.93
	0.50	0.42	-16.58	0.92
	0.80	0.69	-13.19	0.98
$\boldsymbol{\alpha_1^{10}}$	0.20	0.83	312.81	0.92
	0.30	0.18	-40.14	0.95
	0.60	0.53	-12.06	0.95
$\boldsymbol{\alpha_0^{11}}$	-1.50	-1.50	0.11	0.95
	0.90	0.90	0.08	0.96
	0.50	0.50	0.88	0.96
$\boldsymbol{\beta}$	-1.00	-0.98	-1.78	0.78
	0.30	0.29	-2.16	0.83
	0.50	0.49	-1.77	0.85
$\boldsymbol{\gamma}$	-0.80	-0.80	-0.07	0.73
	0.60	0.61	2.37	0.80
	0.40	0.34	-14.13	0.81
τ^2	1.00	1.07	7.26	0.94
σ^2	5.00	4.94	-1.19	0.96
ICC	0.17	0.17	4.40	0.94
**SACE**	**3.37**	**3.11**	**-7.78**	**0.92**
$G = 00$	0.20	0.20	1.08	0.76
$G = 10$	0.29	0.29	0.29	0.77
$G = 11$	0.51	0.50	-0.60	0.67
ϕ^2	0.17	0.11	-32.47	0.71

Web Table 9. Posterior means and coverages for SACE and all model parameters over 200 simulations for $(m, n) = (50,30)$. Simulation data generated with random effects in principal strata model with $\phi^2 = 0.4$, inducing an ICC of 0.10. Model fitted *without* random effects in the principal strata model.

Parameter	True values	Posterior mean	%bias	Coverage
$\boldsymbol{\alpha_1^{11}}$	1.50	1.09	-27.44	0.88
	0.50	0.40	-19.74	0.89
	0.80	0.78	-2.30	0.95
$\boldsymbol{\alpha_1^{10}}$	0.20	0.77	282.52	0.95
	0.30	0.20	-34.49	0.95
	0.60	0.55	-8.07	0.94
$\boldsymbol{\alpha_0^{11}}$	-1.50	-1.47	-1.94	0.95
	0.90	0.90	0.06	0.94
	0.50	0.50	0.90	0.94
$\boldsymbol{\beta}$	-1.00	-0.98	-1.93	0.77
	0.30	0.27	-10.83	0.83
	0.50	0.46	-8.81	0.86
$\boldsymbol{\gamma}$	-0.80	-0.88	10.07	0.69
	0.60	0.60	-0.56	0.83
	0.40	0.36	-10.89	0.89
τ^2	1.00	1.09	9.20	0.95
σ^2	5.00	5.00	0.02	0.94
ICC	0.17	0.17	4.72	0.95
**SACE**	**3.37**	**2.99**	**-11.39**	**0.90**
$G = 00$	0.20	0.21	2.68	0.57
$G = 10$	0.29	0.28	-3.44	0.67
$G = 11$	0.51	0.51	0.91	0.48

Web Table 10. Posterior means and coverages for SACE and all model parameters over 200 simulations for $(m, n) = (50,30)$. Simulation data generated with random effects in principal strata model with $\phi^2 = 0.4$,. inducing an ICC of 0.10. Model fitted with random effects in the principal strata model.

Parameter	True values	Posterior mean	%bias	Coverage
$\boldsymbol{\alpha_1^{11}}$	1.50<br>0.50<br>0.80	1.19<br>0.42<br>0.79	-20.54<br>-15.68<br>-1.01	0.90<br>0.89<br>0.96
$\boldsymbol{\alpha_1^{10}}$	0.20<br>0.30<br>0.60	0.73<br>0.21<br>0.49	265.91<br>-28.74<br>-18.60	0.95<br>0.95<br>0.95
$\boldsymbol{\alpha_0^{11}}$	-1.50<br>0.90<br>0.50	-1.48<br>0.90<br>0.49	-1.31<br>-0.08<br>-1.79	0.96<br>0.95<br>0.95
$\boldsymbol{\beta}$	-1.00<br>0.30<br>0.50	-1.01<br>0.30<br>0.52	0.88<br>0.50<br>4.38	0.71<br>0.86<br>0.83
$\boldsymbol{\gamma}$	-0.80<br>0.60<br>0.40	-0.84<br>0.62<br>0.37	5.08<br>2.78<br>-7.54	0.67<br>0.80<br>0.81
τ^2	1.00	1.11	11.49	0.92
σ^2	5.00	4.98	-0.30	0.95
ICC	0.17	0.18	6.49	0.90
**SACE**	**3.37**	**3.09**	**-8.25**	**0.91**
$G = 00$<br>$G = 10$<br>$G = 11$	0.20<br>0.29<br>0.51	0.20<br>0.29<br>0.51	1.57<br>-1.03<br>-0.03	0.68<br>0.74<br>0.66
ϕ^2	0.40	0.43	7.20	0.92

Web Table 11. Posterior means and coverages for SACE and all model parameters over 200 simulations for $(m, n) = (75,20)$.

**Parameter**	**True values**	**Posterior mean**	**%bias**	**Coverage**
$\boldsymbol{\alpha_1^{11}}$	1.50	1.33	-11.18	0.96
	0.50	0.46	-7.57	0.93
	0.80	0.77	-3.35	0.91
$\boldsymbol{\alpha_1^{10}}$	0.20	0.56	179.90	0.94
	0.30	0.24	-19.08	0.95
	0.60	0.55	-7.87	0.95
$\boldsymbol{\alpha_0^{11}}$	-1.50	-1.49	-0.80	0.95
	0.90	0.90	0.31	0.95
	0.50	0.50	0.13	0.95
$\boldsymbol{\beta}$	-1.00	-1.01	0.64	0.62
	0.30	0.30	1.38	0.63
	0.50	0.51	1.19	0.69
$\boldsymbol{\gamma}$	-0.80	-0.80	-0.21	0.68
	0.60	0.60	0.39	0.66
	0.40	0.40	1.11	0.64
τ^2	1.00	1.13	13.42	0.93
σ^2	5.00	4.97	-0.64	0.95
ICC	0.17	0.18	7.03	0.94
**SACE**	**2.85**	**2.73**	**-4.34**	**0.95**
$G = 00$	0.21	0.21	-0.25	0.76
$G = 10$	0.26	0.26	-0.06	0.84
$G = 11$	0.45	0.52	0.13	0.72

Web Table 12. Posterior means and coverages for SACE and all model parameters over 200 simulations for $(m, n) = (25, 20)$.

Parameter	True values	Posterior mean	%bias	Coverage
$\boldsymbol{\alpha_1^{11}}$	1.50	1.10	-26.51	0.92
	0.50	0.43	-14.13	0.96
	0.80	0.73	-8.71	0.94
$\boldsymbol{\alpha_1^{10}}$	0.20	0.87	336.63	0.97
	0.30	0.17	-43.38	0.96
	0.60	0.53	-11.46	0.99
$\boldsymbol{\alpha_0^{11}}$	-1.50	-1.51	0.53	0.93
	0.90	0.90	0.05	0.96
	0.50	0.50	0.90	0.99
$\boldsymbol{\beta}$	-1.00	-1.04	3.78	0.92
	0.30	0.31	4.35	0.95
	0.50	0.51	2.51	0.93
$\boldsymbol{\gamma}$	-0.80	-1.00	24.63	0.87
	0.60	0.67	11.26	0.89
	0.40	0.42	5.59	0.89
τ^2	1.00	1.16	16.35	0.91
σ^2	5.00	4.92	-1.64	0.97
ICC	0.17	0.18	8.38	0.92
**SACE**	**2.85**	**2.60**	**-8.89**	**0.94**
$G = 00$	0.21	0.22	2.53	0.76
$G = 10$	0.26	0.25	-4.44	0.88
$G = 11$	0.52	0.53	1.22	0.75

Web Table 13. Posterior means and coverages for SACE and all model parameters over 200 simulations for $(m,n) = (30,50)$. The induced outcome ICC is 0.05.

**Parameter**	**True values**	**Posterior mean**	**%bias**	**Coverage**
$\boldsymbol{\alpha_1^{11}}$	1.50	1.29	-14.24	0.93
	0.50	0.45	-9.92	0.93
	0.80	0.77	-4.09	0.94
$\boldsymbol{\alpha_1^{10}}$	0.20	0.60	201.59	0.96
	0.30	0.22	-25.50	0.96
	0.60	0.56	-6.78	0.96
$\boldsymbol{\alpha_0^{11}}$	-1.50	-1.52	1.16	0.96
	0.90	0.90	-0.39	0.94
	0.50	0.49	-1.62	0.96
$\boldsymbol{\beta}$	-1.00	-1.01	1.35	0.58
	0.30	0.30	-0.49	0.59
	0.50	0.51	1.50	0.60
$\boldsymbol{\gamma}$	-0.80	-0.81	0.87	0.62
	0.60	0.59	-0.83	0.63
	0.40	0.40	0.81	0.64
τ^2	0.26	0.27	3.13	0.89
σ^2	5.00	4.99	-0.14	0.94
ICC	0.05	0.05	1.62	0.88
**SACE**	**2.85**	**2.70**	**-5.05**	**0.96**
$G = 00$	0.18	0.21	-0.06	0.67
$G = 10$	0.26	0.27	0.11	0.79
$G = 11$	0.45	0.52	-0.03	0.66

Web Table 14. Posterior means and coverages for SACE and all model parameters over 200 simulations for $(m, n) = (30, 50)$. The induced outcome ICC is 0.01.

Parameter	True values	Posterior mean	%bias	Coverage
$\boldsymbol{\alpha_1^{11}}$	1.50<br>0.50<br>0.80	1.30<br>0.46<br>0.77	-13.27<br>-8.55<br>-3.46	0.95<br>0.92<br>0.95
$\boldsymbol{\alpha_1^{10}}$	0.20<br>0.30<br>0.60	0.60<br>0.22<br>0.55	199.71<br>-26.89<br>-8.26	0.94<br>0.96<br>0.95
$\boldsymbol{\alpha_0^{11}}$	-1.50<br>0.90<br>0.50	-1.50<br>0.89<br>0.50	0.08<br>-0.87<br>-0.46	0.96<br>0.93<br>0.95
$\boldsymbol{\beta}$	-1.00<br>0.30<br>0.50	-1.01<br>0.31<br>0.51	1.27<br>3.02<br>1.05	0.58<br>0.59<br>0.58
$\boldsymbol{\gamma}$	-0.80<br>0.60<br>0.40	-0.81<br>0.60<br>0.41	1.67<br>0.82<br>1.45	0.61<br>0.61<br>0.61
τ^2	0.05	0.05	-4.37	0.99
σ^2	5.00	4.99	-0.19	0.99
ICC	0.01	0.01	-4.70	0.99
**SACE**	**2.85**	**2.70**	**-5.23**	**0.91**
$G = 00$<br>$G = 10$<br>$G = 11$	0.18<br>0.26<br>0.45	0.21<br>0.27<br>0.52	-0.32<br>0.10<br>0.08	0.69<br>0.82<br>0.64

Web Table 15. Descriptive statistics for always-survivors by intervention arms based on Bayesian posterior sample for principal strata membership.

**Covariates**	**Intervention**	**Control**	**Total**
Gender			
Female	173(35.1%)	187(33.6%)	360(34.3%)
Male	320(64.9%)	370(66.4%)	690(65.7%)
Age	73.4(14.4)	73.5(13.7)	73.4(14.0)
Ethnicity			
White	429(87.0%)	491(88.2%)	920(87.6%)
Non-white	64(13.0%)	66(11.8%)	130(12.4%)
Highest level of education			
No formal education	313(63.5%)	363(65.2%)	676(64.4%)
GCSE/ O'levels	88(17.8%)	120(21.5%)	208(19.8%)
A'levels/HNC	25(5.1%)	38(6.8%)	63(6.0%)
University level	24(4.8%)	12(2.2%)	36(3.4%)
Grad or professional	44(8.9%)	24(4.3%)	68(6.5%)
Only adult household			
Yes	261(53.4%)	254(54.4%)	564(53.7%)
No	233(46.6%)	303(45.6%)	487(46.3%)
Number of comorbidities	1.0(1.4)	1.1(1.4)	1.1(1.4)
Impairment score	28.2(14.5)	28.9(15.7)	28.6(15.1)
Physical health score	28.4(8.5)	27.9(8.6)	28.1(8.6)
Mental health score	33.2(7.9)	33.1(7.8)	33.2(7.9)
EQ-5D-VAS index score	52.9(21.8)	53.3(22.0)	53.1(21.9)
